# Idiopathic Aqueductal Stenosis: Late Neurocognitive Outcome in ETV Operated Adult Patients

**DOI:** 10.3389/fneur.2022.806885

**Published:** 2022-04-07

**Authors:** Matteo Martinoni, Giovanni Miccoli, Luca Albini Riccioli, Francesca Santoro, Giacomo Bertolini, Corrado Zenesini, Diego Mazzatenta, Alfredo Conti, Luigi Maria Cavallo, Giorgio Palandri

**Affiliations:** ^1^IRCCS Istituto delle Scienze Neurologiche di Bologna, Bologna, Italy; ^2^Department of Neuroscience and Reproductive and Odontostomatological Sciences, University of Naples Federico II, Naples, Italy; ^3^Neuroradiology Unit, IRCCS Istituto delle Scienze Neurologiche di Bologna, Bologna, Italy; ^4^Neurology Unit, IRCCS Istituto delle Scienze Neurologiche di Bologna, Bologna, Italy; ^5^Epidemiology and Biostatistics Service, IRCCS Istituto delle Scienze Neurologiche di Bologna, Bologna, Italy

**Keywords:** aqueductal stenosis, hydrocephalus, endoscopic third ventriculostomy, LIAS, chronic adult hydrocephalus, late onset hydrocephalus

## Abstract

**Objective:**

The aim of the present study is to evaluate a neurocognitive outcome in patients affected by late-onset idiopathic aqueductal stenosis (LIAS) who underwent endoscopic third ventriculostomy (ETV).

**Materials and Methods:**

A prospective study was conducted between January 2015 and December 2017 in a series of 10 consecutive adult patients referred to the Neurosurgery Department of IRCCS Istituto delle Scienze Neurologiche di Bologna, Bologna, Italy. All the adult patients admitted with absence of CSF flow through the aqueduct in phase-contrast (PC)—MRI sequences or a turbulence void signal in T2—weighted images in midsagittal thin-slice MR sequences underwent a specific neuroradiological, neurological, and neurocognitive assessment pre- and postoperatively.

**Results:**

All patients affected by gait and sphincter disturbances improved after ETV. Attentive and executive functions as well as visuo-spatial memory and verbal executive functions improved in several patients. Similarly, the affective and behavioral scales improved in almost 50% of the patients. No major complications have been recorded, and no patients required a second surgery for shunt placement.

**Conclusion:**

Endoscopic third ventriculostomy represents a safe and effective surgical procedure for the treatment of LIAS. In addition to neurological improvement, we demonstrated also postoperative neurocognitive improvement mainly in attentive and executive functions, visuo-spatial memory, verbal executive functions, and behavioral and affective domains.

## Introduction

Aqueductal stenosis (AS) is a cause of obstructive hydrocephalus whose clinical presentation occurs mainly during childhood and adolescence. In the adult population, it represents about 10% of all types of hydrocephalus (1–6). AS may have several etiologies: extrinsic compression from tumoral lesions, genetic disorders, post infectious, post hemorrhagic (1, 7–11). Idiopathic AS (IAS) consists of intrinsic congenital or acquired obstruction of the CSF pathway in the Sylvian aqueduct (12, 13). Late-onset idiopathic aqueductal stenosis (LIAS) is a clinical entity radiologically defined as a non-communicating triventricular hydrocephalus with idiopathic obstruction at the level of the cerebral aqueduct manifesting in adult age (6, 14). LIAS usually mimics normal pressure hydrocephalus symptoms (cognitive impairment, gait disturbances, and urinary incontinence), sometimes coexisting with obstructive hydrocephalus manifestations related to an increase of intracranial pressure (ICP) (e.g., endocrinological/visual/ocular disturbances, extrapyramidal signs, headache, etc.) (6).

Endoscopic third ventriculostomy (ETV) currently represents the gold standard treatment of LIAS (4, 6, 12). To date, validated scales are available to predict the surgical success and failure of ETV (7, 15–17) in AS, such as the ETV success score, that, although firstly developed for pediatric population, found its validations also among adults' cases (15). Anyway, there is still sparse literature on neurocognitive results (4, 6, 18, 19).

The aim of the present study is to evaluate the neurocognitive outcome in patients with LIAS who underwent ETV.

## Materials and Methods

A prospective study was conducted between January 2015 and December 2017 at the Neurosurgery Department of IRCCS. Istituto delle Scienze Neurologiche di Bologna, Bologna, Italy. The study protocol was approved by the Local Ethics Committee of the local health service of Bologna, Italy (Cod. CE: 14131, 23/02/2015).

Inclusion criteria were as follows: patients aged 18 years or over; triventricular hydrocephalus with absence of CSF flow through the aqueduct in phase contrast (PC)—MRI sequences or a turbulence void signal in T2—weighted images in midsagittal thin-slice MR sequences; patients having undergone an ETV procedure. Exclusion criteria were: intracerebral tumors, history of intracranial bleeding or infection, previous cerebral surgeries, psychiatric diseases, major neurological diseases/therapies (e.g., epileptic patients with or without antiepileptic drugs).

All the recruited patients received neurocognitive evaluation pre- and postoperatively.

All the data have been prospectively recorded since the admission.

### Neuroradiological Protocol

MRI was performed on a 3 Tesla whole-body scanner (Magnetom Skyra, Siemens Healthcare, Erlangen, Germany) using a 32-channel phased-array head coil. A sagittal and axial T1 WI sequence, a sagittal and axial T2 WI sequence, an axial FLAIR sequence, a 3D sagittal T1WI (1 mm) sequence, a turbo spin-echo T2 flow (3 mm) sequence, and a PC-MRI sequence were performed. The following neuroradiological features for every patient were evaluated: Evans' index (<0.25/0.25 < EI <0.3/>0.3); narrow sulci (normal, parafalcine, vertex); Sylvian fissure enlargement; focally enlarged sulci; widened temporal horns (<0.4/0.4 < TH <0.6/>0.6); a callosal angle (>90°/60° < CA <90°/ <60°); Fazekas scale for white matter lesions; Fazekas scale; age-related white matter changes (ARWMC) (20); height of an interpeduncular cistern (and related third ventricle's bulging).

In order to assess dilation occurred in posterior segments of the lateral ventricle, the fronto-occipital horn ratio (FOHR) was indicated, when needed, to overcome such limitation of Evan's index.

All MRI exams were evaluated pre- and postoperatively by the same neuroradiologist (L.A.R.) who was blind to the neurological and neurocognitive assessment.

### Neurological Protocol

The following neurological features were evaluated according to the iNPH grading score according to Kubo et al. (21), evaluating gait, sphincter, and cognitive function. The score of each domain ranges from 0 to 4, with higher scores indicating worse symptoms. The presence of a preoperative headache was recorded (Yes/No). Time of onset/duration of clinical signs and symptoms before being admitted and operated on was recorded according to Fukuhara's classification (6): Class I (symptom duration under 1 month), Class II (symptom duration from 1 to 6 months), Class III (symptom duration over 6 months).

### Neurocognitive Protocol

A tailored neurocognitive evaluation was assessed before and after ETV by a dedicated neuropsychologist and with a universally recognized questionnaire. The following neurocognitive functions were evaluated with the associated tests: general screening (mini mental state examination); attentional and executive functions (attentional matrices, Trail Making A, Trail Making B, Trail Making A-B, Stroop test—error, Stroop test—time); logical-abstract reasoning (Raven's colored progressive matrices in mental deterioration battery); visuo-perceptive and visuo-constructive abilities (Montreal cognitive Rey-Osterrieth complex figure test-−10' recall); visuo-spatial memory (Corsi test, Supra-Span Corsi test, Montreal cognitive Rey-Osterrieth complex figure test-−10' recall); verbal memory (a digit span—forward, a Rey 15-word immediate recall, a Rey 15-word late recall, a Babcock story recall test); language and verbal executive functions (phonemic word fluency test in mental deterioration battery, semantic fluency); functional scales (activities of daily living, instrumental activities of daily living); affective and behavioral scales (Beck depression inventory—affective and somatic, Beck depression inventory—cognitive, Beck depression inventory—total, state-trait anxiety inventory Y1—state, state-trait anxiety inventory Y2—trait).

Individual scores of the different tests were calculated using the equivalent scores (ES) in order to correct the subject's raw score, eliminating the influence of age and education. For each test, the adjusted scores are then transferred to a 5-level scale (0–4). The ES represent a method of correcting neuropsychological tests. This method of non-parametric correction of the ES was devised by Capitani (22) and colleagues in the context of an Italian multicentric study on the calibration of neuropsychological tests (23) still today a cornerstone for neuropsychological evaluation. The Italian neuropsychological tests were constructed according to the methodology-described use—after the correction of the raw score for sex, age, and education—a system of scores on an ordinal scale, called equivalent scores, ranging from 0 to 4, corresponding to as many segments of the distribution [0 = deficient, 1 = border line, 2 and 3 = middle inferior (between 20 and 50 percentiles), and 4, middle superior (over 50 percentile)]

Time between the first neuropsychological evaluation and intervention ranged from 1 to 65 days (mean, 17.4). Time between surgery and the second neuropsychological assessment was from 120 to 425 days (mean, 258.5).

We considered a test as improved when the postoperative score showed an increase of at least two units or only one if the initial score was 0 (pathological condition). A worse result was defined by a decrease of two units or only one if it reaches the pathological condition (0). In the same way, for BDI and STAI Y tests, the cut-off was, respectively, the 85th and 40th percentiles, and a patient was considered improved if the postoperative score was lower than or equal to these percentiles as compared to preoperatively higher values (24). The comparison was made according to Ghisi et al.

All neuropsychological examinations were conducted by the same neuropsychologist (F.S.).

### Surgical Procedure

The procedure is performed under general anesthesia. Through a burr hole drilled 0.5 cm in front of the coronal suture and 2.5 cm lateral from the midline on the right side, the right lateral ventricle is cannulated using a 14F peel-away catheter with a blunt-tipped obturator. A rigid rod lens endoscope (Karl Storz LOTTA System®, 6° angle) is then inserted. The anatomical landmarks of the lateral ventricle (choroid plexus, thalamostriate, and septal veins, foramen of Monro) are identified. Through the foramen of Monro, the third ventricle is accessed with the endoscope recognizing the mammillary bodies and the infundibular recess. The standard target lies in the tuber cinereum, (e.g., the region of the third ventricle floor between the infundibular recess and the mammillary bodies, that is usually perforated with blunt-closed Decq forceps. The stoma is dilated by inflating a Fogarty catheter. Thereafter, the underlying subarachnoid space is explored with the endoscope. The clear vision of the structures of the interpeduncular cistern (the basilar artery and/or its branches, brainstem, third cranial nerve, and dura of the clivus) is the goal of surgery. The pulsation of the third ventricular floor, and mainly of the edges of the stoma, is an indirect marker of the CSF flowing through the performed stoma. All ETV procedures were performed by the same group of neuroendoscopy surgeons (G.P. and M.M).

### Statistical Analysis

In the descriptive analysis, results were presented as median and interquartile range (IQR). The comparison of neuropsychological status before and after surgery was evaluated with the non-parametric Wilcoxon signed-rank test. Statistical analysis was performed using statistical package Stata SE, 14.2.

## Results

Fifteen patients initially met the inclusion criteria: three were excluded because of secondary aqueductal stenosis, one for a previous surgery, and one since affected by epilepsy (on anti-epileptic drug treatment). Finally, 10 consecutive patients (5 males and 5 females) with a mean age of 50.5 years (23–73 years) were included in the study. Patients' follow-up ranged from 14 to 36 months (mean follow-up, 24.9).

No surgical and clinical intra- or postoperative complications were reported. No patients required further surgery (ETV or shunt placement).

### Clinical Status

According to Fukuhara's classification, eight patients were referred with chronic symptomatology (over 6 months), one with subacute symptomatology (between 1 and 6 months), while one patient was not able to recall detailed data on onset/duration of his clinical conditions. Two patients complained of headache preoperatively, while no one was still affected once operated on. According to the iNPH grading scale, preoperatively, eight patients had gait impairment, six patients had sphincter impairment, and six patients had cognitive impairment. During the postoperative period, all the patients affected by gait and sphincter disturbances improved. Four out of the six patients with preoperative cognitive impairment improved; two had no changes. No clinical worsening was reported (All clinical data are reported in Table 1).

**Table 1:** Pre- and post-operative clinical evaluation—iNPH grading score (21) and Fukuhara (6) classification.

**PT**	**Gait**		**Sphincter**		**Cognitive**		**iNPH total**		**Headache**		**Fukuhara**
**Patients**	**Pre**	**Post**	**Pre**	**Post**	**Pre**	**Post**	**Pre**	**Post**	**Pre**	**Post**	
1	2	0	1	0	1	0	4	0	N	N	III
2	1	0	0	0	0	0	1	0	Y	N	III
3	2	0	1	0	NA	0	3	0	N	N	III
4	0	0	0	0	0	0	0	0	N	N	NA
5	2	0	0	0	2	1	4	1	N	N	III
6	0	0	0	0	0	0	0	0	Y	N	II
7	2	0	2	0	2	0	6	0	N	N	III
8	2	0	1	0	1	1	4	1	N	N	III
9	2	1	2	0	2	1	6	2	N	N	III
10	2	0	2	0	2	2	6	2	N	N	III

*PT, Patient; iNPH, idiopathic Normal Pressure Hydrocephalus score*.

### Neuroradiological Findings

**Figure 1:**
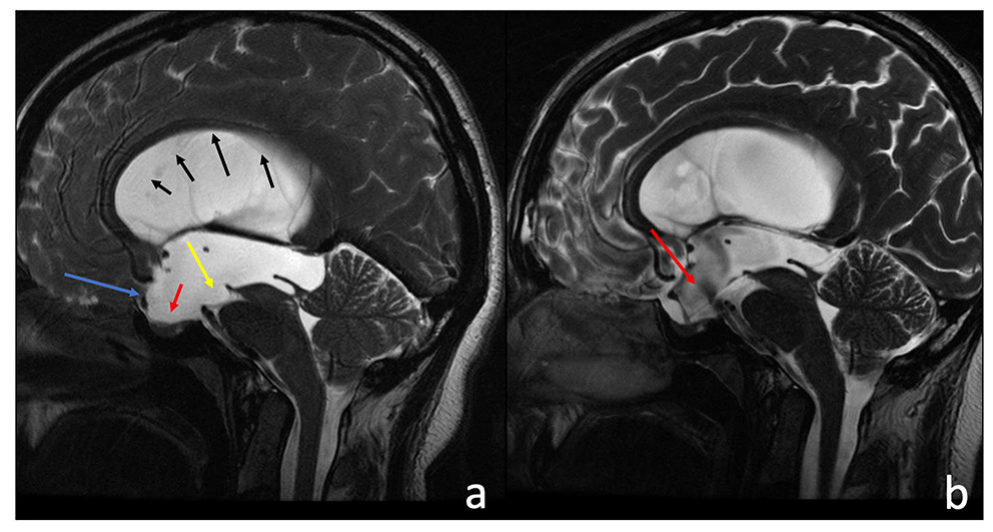
Sagittal T2-w 3D-FIESTA acquisition of a 38-year-old male before **(a)** and after **(b)** third ventriculostomy. **(a)** The third ventricle and the lamina terminalis are concave (**a**, blue arrow), the sella turcica is empty (**a**, red arrow), and there is an evident compressive effect exerted on the brain stem and the ambiens cistern (**a**, yellow arrow). The corpus callosum appears thinned (black sequential arrows), and subarachnoid spaces have a low representation. After the third ventriculostomy **(b)**, there are evident flow artifacts at the floor of the III ventricle (**a**, red arrow). All previous neuroradiological findings clearly improved.

**Figure 2:**
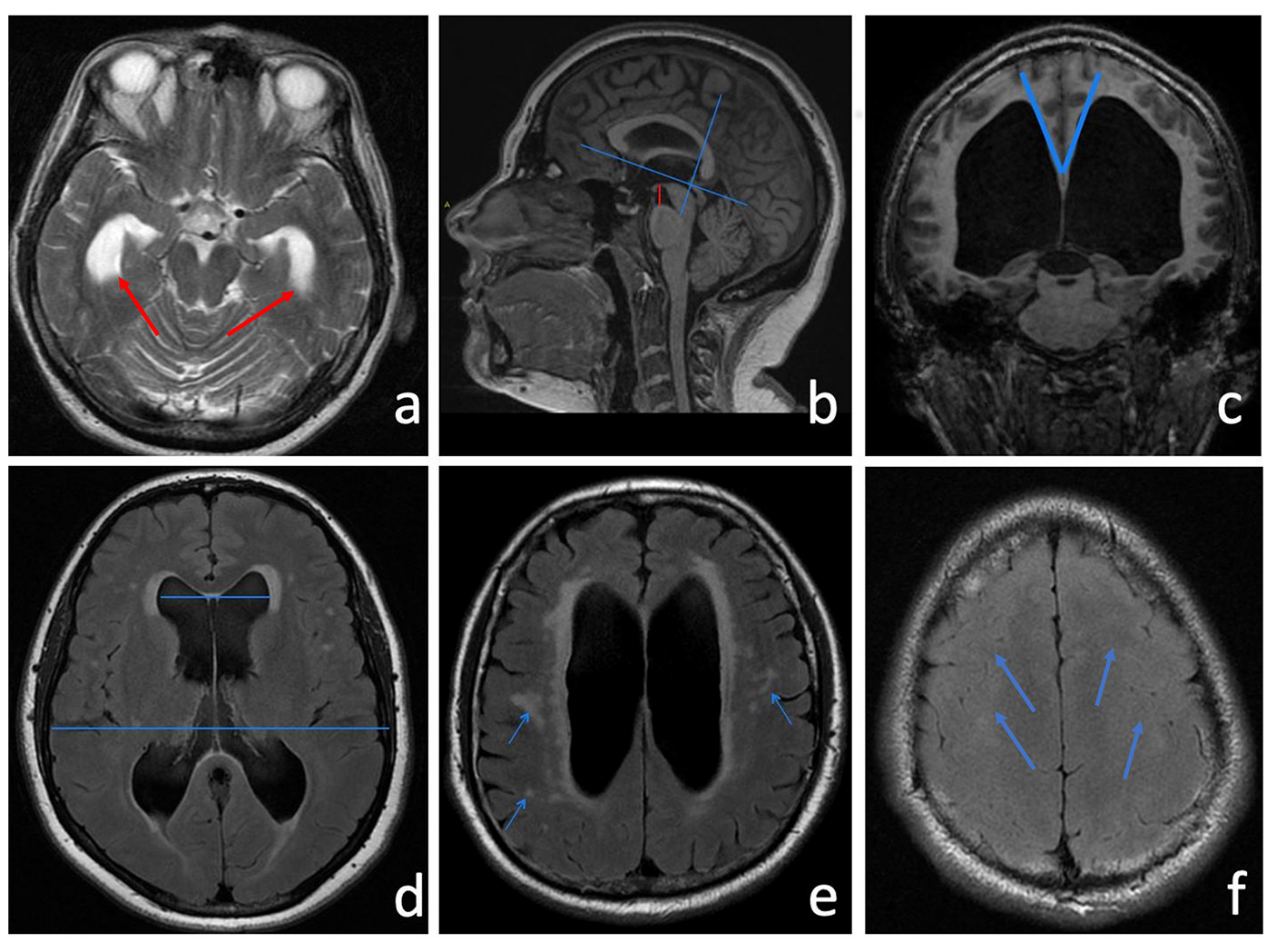
**(a)** Axial T2-w showing a clear enlargement of the temporal horns (red arrows). **(b)** Sagittal 3D MPRAGE T1-w was used to measure the height of the interpeduncular cistern, which represents the shortest distance between the floor of the third ventricle and the midbrain (a red line). After identifying the AC-PC plane, the callosal angle **(c)** is measured in the coronal plane through the posterior commissure perpendicular to the anterior commissure-posterior commissure (AC-PC) plane. Neuroradiological features of LIAS mimic iNPH also for CA amplitude that usually measures <90°. **(d)** Evan's index (EI) is a direct linear measurement of the ventricular size. It is calculated from the ratio between the maximum transverse diameter of the frontal horns and the maximum internal diameter of the skull. **(e)** The FLAIR sequence shows an elevated periventricular signal ascribable to interstitial edema or reactive gliosis. Hyperintense white matter lesions attributed to chronic ischemia of the small vessels are indicated by the arrows. **(f)** Narrowed sulci at the high cerebral convexities (blue arrows).

All patients had a preoperative Evans' index >0.3. Preoperatively, all patients' MRI showed third ventricle's bulging. After ETV, two patients reported a reduction of Evans' index between 0.25 and 0.3; the others were stable. One patient had a preoperative callosal angle >90°, seven patients had a callosal angle between 60° and 90°, and two patients had a callosal angle <60°. After ETV, the callosal angle widened in five patients, while it was unchanged in the other five. Four patients had a preoperative Fazekas score of 0, one patient of 1, one patient of 2, one patient of 3, one patient of 4, and two patients of 5. After ETV, three patients improved, while seven were stable. The preoperative median value of interpeduncular cistern height was 5.75 mm (average, 6.14 mm; range, 3.7–9.6 mm), while, after ETV, the median value of interpeduncular cistern height was 7.7 mm (average, 8.01 mm; range, 5.2–10.5 mm). No neuroradiological worsening was observed. Postoperative T2WI in midsagittal thin slice confirmed the flow void through the stoma in all patients (All neuroradiological data were reported in Table 2; Figures 1, 2).

**Table 2:** Pre- and postoperative neuroradiological evaluation.

**Pt**	**Evan's Index**		**Narrowed Sulci**		**Sylvian fissure**		**Focally enlarged sulci**		**Temporal Horns**		**Callosal Angle**		**Fazekas DWM**		**Fazekas**		**ARWMC**		**Total**		**Interped.Cystern**	
	**Pre**	**Post**	**Pre**	**Post**	**Pre**	**Post**	**Pre**	**Post**	**Pre**	**Post**	**Pre**	**Post**	**Pre**	**Post**	**Pre**	**Post**	**Pre**	**Post**	**Pre**	**Post**	**Pre**	**Post**
1	2	2	2	0	0	0	1	1	2	1	1	1	2	2	5	4	7	6	22	17	8.4	9.1
2	2	1	0	0	0	0	0	0	1	0	0	0	0	0	0	0	0	0	3	1	9.6	10.5
3	2	2	2	0	0	0	0	0	2	2	2	1	1	0	2	0	0	0	11	5	3.7	7.7
4	2	2	1	0	0	0	0	0	2	1	1	0	0	0	0	0	0	0	6	3	7.4	9.7
5	2	2	1	0	0	0	0	0	2	0	1	0	0	0	0	0	0	0	6	2	7.4	9.9
6	2	1	2	1	0	0	0	0	2	1	1	0	1	1	3	3	4	4	15	11	5.9	7.7
7	2	2	2	0	0	0	0	0	2	2	2	2	0	0	0	0	0	0	8	6	5.02	7.24
8	2	2	0	0	0	0	0	0	2	2	1	0	2	2	4	4	10	10	21	20	3.9	6.2
9	2	2	2	0	0	0	0	0	2	1	1	1	2	2	5	4	8	8	22	19	5.6	6.9
10	2	2	0	0	0	0	0	0	2	2	1	1	1	1	1	1	0	0	7	7	4.5	5.2

*PT, Patient; Fazekas DWM, Fazekas score for white matter lesions; ARWMC, age-related white matter changes*.

### Neuropsychological Status

Neurocognitive domains were carefully evaluated pre and post ETV as follows:

- MMSE: rather surprisingly, it appears normal in all patients. This finding is not typical for late-onset hydrocephalus.
- Attentive and executive function tests: two out of 10 patients improved in the attentional matrices test (*p* = 0.087); three had best results in the trail making type A test, one patient worsened; two patients showed higher scores at the final follow-up in the trail making type B test (*p* = 0.010), and three patients in the trail making B-A test (*p* = 0.010); one patient improved in Stroop test error once operated on, while one worsened; three patients recovered post ETV in Stroop test time, only two worsened.
- Logical abstract reasoning pointed out worsened results after ETV in two patients (Raven's colored progressive matrices in the mental deterioration battery test); a better result was collected in one case.
- Visual-perceptive and visual-constructive skills: one patient improved, while one worsened in the Rey–Osterrieth complex figure test.
- Visuo-spatial memory domain: five patients improved in the Corsi test, while one patient worsened at the final follow-up; four patients improved in the Corsi Supra span test; three patients of the series results were better in Rey's recall test, while one patient showed a new deficiency after surgery.
- Verbal memory field: four patients lowered their performances postoperatively in the digit span test; two patients improved, while two worsened their results in the 15-word Rey immediate test, only one patient successfully improved his preoperative results in the delayed type of the latter test; three patients finally improved in Babcock story recall test in the postoperative period.
- Language and verbal executive functions: three patients improved on phonemic word fluency (*p* = 0.047); one patient showed lower scores after ETV; a test exploring semantic fluency showed improvement after the surgical procedure in four patients.

The functional scale used to assess activities of daily living (ADL) highlighted positive changes for one patient; the instrumental activities of daily living (IADL) scale showed better scores for three and worse for two patients at the last follow-up.

Affective and behavioral domains were examined: two patients improved the results in somato-affective BDI (*p* = 0.011) compared to the preoperative period and three in the cognitive BDI, only one patient worsened his results in cognitive BDI after ETV. The total BDI score improved in three patients (< /=85th percentile) compared to preoperative levels, while it was over the cut-off in one patient.

Three patients improved their results in the state-trait inventory Y1 test (state) (*p* = 0.006) and three in the state-trait inventory Y2 test (trait) (below the 40th percentile) (*p* = 0.041) (Neuropsychological domains improved after ETV are shown in Table 3; neuropsychological comparison before and after surgery with statistical analysis is presented in Table 4; all neuropsychological data are reported in even more detail in Table 5).

**Table 3:** neuropsychological status of patients after ETV, herein in green are signaled the neuropsychological domains improved with statistical significance.

**Neuropsychological status post ETV**		
**Domains**	**Patients improved**	**Patients worsened**
Attentional and executive functions	6	3
Logical-abstract reasoning	1	2
Visuo-perceptive and visuo-constructive abilities	1	1
Visuo-spatial memory	7	2
Verbal memory	5	4
Language and verbal executive functions	5	1
Functional scales	3	2
Affective and behavioral scales	6	1

**Table 4:** Comparison of neuropsychological status before and after ETV.

	**Before Median (IQR)**	**After Median (IQR)**	**Differences Before-After**	***p*-value Wilcoxon**
MMSE	27.8 (27.5–28.9)	28.2 (26.8–28.8)	5 +, 5 =, 0 -	0.644
Matrices test	3.5 (2–4)	4 (4–4)	5 +, 4 =, 1 -	0.087
Trail making A	4 (2–4)	4 (4–4)	3 +, 6 =, 1 -	0.292
Trail making B	3 (1–4)	4 (4–4)	7 +, 3 =, 0 -	0.010*
Trail making B-A	3 (1–4)	4 (4–4)	7 +, 3 =, 0 -	0.010*
Stroop test error	4 (4–4)	4 (4–4)	2 +, 7 =, 1 -	0.565
Stroop test time	4 (2–4)	4 (3–4)	4 +, 4 =, 2 -	0.423
Raven's matrices	4 (4–4)	4 (4–4)	2 +, 6 =, 2 -	0.861
Rey–Osterrieth	4 (3–4)	4 (3–4)	2 +, 6 =, 2 -	0.954
Corsi test	1.5 (0–4)	3 (1–4)	5 +, 4 =, 1 -	0.123
Corsi Supraspan	2 (1–4)	4 (3–4)	5 +, 3 =, 2 -	0.131
Delayed Recall	1.5 (0–3)	2.5 (1–4)	4 +, 3 =, 3 -	0.403
Digit Span test	3.5 (2–4)	2.5 (1–4)	2 +, 3 =, 5 -	0.129
Words Rey Imm	2 (0–4)	2 (1–3)	5 +, 2 =, 3 -	0.679
Words Rey Diff	1 (0–3)	2 (0–4)	2 +, 6 =, 2 -	0.907
Babcock story recall test	2 (2–2)	2.5 (2–3)	5 +, 2 =, 3 -	0.252
Semantic fluency	0 (0–3)	3 (0–4)	4 +, 5 =, 1 -	0.151
Phonemic fluency	2.5 (0–4)	3.5 (2–4)	4 +, 6 =, 0 -	0.047*
ADL	6 (6–6)	6 (6–6)	1 +, 9 =, 0 -	0.317
IADL	5 (5–8)	5 (5–8)	3 +, 5 =, 2 -	0.659
BDI SA	70 (50–99)	55 (20–80)	7 +, 3 =, 0 -	0.011*
BDI C	75 (60–85)	65 (50–70)	5 +, 3 =, 2 -	0.232
BDI total	65 (50–96)	55 (20–80)	5 +, 3 =, 2 -	0.132
STAI Y State	43.5 (38–46)	32.5 (29–41)	9 +, 1 =, 0 -	0.006*
STAI Y Trait	44.5 (39–52)	33 (32–42)	7 +, 0 =, 3 -	0.041*

*p <0.05: statistical evidence; + improvement, = no differences, - worsening; MMSE, mini-mental state examination; ADL, Activities of Daily Living; IADL, Instrumental activities of daily living; BDI SA, Beck Depression Inventory affective and somatic; BDI C, Beck Depression Inventory cognitive; STAI State/Trait, State-Trait Anxiety Inventory State/State-Trait Anxiety Inventory Trait*.

**Table 5:** Detailed description of neuropsychological pre- and postoperative evaluation tests used.

**Test**	**PT1**		**PT2**		**PT3**		**PT4**		**PT5**		**PT6**		**PT7**		**PT8**		**PT9**		**PT10**	
	**Pre**	**Post**	**Pre**	**Post**	**Pre**	**Post**	**Pre**	**Post**	**Pre**	**Post**	**Pre**	**Post**	**Pre**	**Post**	**Pre**	**Post**	**Pre**	**Post**	**Pre**	**Post**
**General screening**																				
Mini-Mental state examination	27,49	26,49	30	28,59	25,59	27,59	27,75	28,75	27,49	28,49	28,89	27,89	27,79	30	25,85	25,44	28,88	30	28,73	26,8
**Attentional and executive functions**																				
Attentional matrices	4	3	3	4	4	4	4	4	4	4	2	3	3	4	1	4	4	4	2	4
Trail Making A	4	4	4	4	2	4	4	4	2	4	3	1	4	4	4	4	4	4	1	4
Trail Making B	1	2	3	4	3	4	4	4	1	4	1	2	4	4	3	4	4	4	2	4
Trail Making B-A	0	1	3	4	3	4	4	4	1	4	1	2	4	4	3	4	4	4	2	4
Stroop test—error	4	4	4	4	4	4	4	4	4	4	2	3	4	4	1	4	4	4	4	2
Stroop test—time	2	4	2	4	4	4	4	4	0	4	2	3	4	4	4	2	4	4	4	2
**Logical-abstract reasoning**																				
Raven's Colored Progressive Matrices in Mental Deterioration Battery	4	4	4	4	4	4	4	4	3	4	4	2	4	4	4	4	4	0	2	4
**Visuo-perceptive and visuo-constructive abilities**																				
Rey–Osterrieth complex figure test—copy	2	4	4	4	4	4	4	3	4	4	4	1	3	4	4	4	4	4	0	0
**Visuo-spatial memory**																				
Corsi test	0	1	3	3	0	3	4	1	0	4	4	4	0	3	1	1	2	4	4	4
Supra-Span Corsi test	3	4	4	4	1	3	4	3	0	4	4	3	0	0	1	4	4	4	1	4
Montreal cognitive Rey-Osterrieth complex figure test-−10' recall	1	3	4	4	1	0	3	4	0	4	3	2	2	1	0	2	4	4	0	0
**Verbal memory**																				
Digit span—forward	3	4	4	4	4	3	4	2	1	1	2	0	2	3	2	0	4	2	4	4
Rey 15-word—immediate recall (IR) ability	1	2	4	3	3	1	3	4	0	3	4	0	0	0	1	2	4	4	0	2
Rey 15-word—delayed recall (DR) ability	2	1	3	3	0	0	3	4	0	4	4	3	0	0	0	0	4	4	0	0
Babcock story recall test	2	2	4	3	2	3	2	4	0	3	3	2	2	1	1	2	2	4	2	2
**Language and verbal executive functions**																				
Phonemic word fluency test in Mental Deterioration Battery	0	3	3	1	4	4	4	4	0	3	0	0	0	0	0	0	3	4	0	3
Semantic fluency	3	3	4	4	3	3	4	4	0	4	0	1	2	4	2	2	4	4	0	2
**Functional scales**																				
Activities of daily living	6	6	6	6	6	6	6	6	4	6	6	6	6	6	6	6	5	5	6	6
Instrumental activities of daily living	5	5	5	5	8	5	8	8	4	8	8	5	5	8	5	5	6	8	5	5
**Affective and behavioral scales**																				
Beck Depression Inventory—affective and somatic (cut- off > 85°)	85	85	99	80	70	60	20	20	99	20	50	20	50	20	50	50	70	60	99	90
Beck Depression Inventory—cognitive (cut- off > 85°)	70	70	99	70	70	60	40	40	99	40	60	60	80	50	50	70	80	99	85	70
Beck Depression Inventory—total (cut- off > 85°)	80	80	99	70	60	60	20	20	99	20	50	20	60	20	40	50	70	90	96	85
State-Trait Anxiety Inventory Y1—state (cut-off >40)	37	36	46	33	36	29	38	31	44	41	39	21	44	27	43	32	48	45	52	52
State-Trait Anxiety Inventory Y2—trait (cut-off >40)	46	42	57	32	39	33	38	31	61	41	42	32	43	33	27	32	52	56	49	51

*We considered a test as improved when the postoperative score showed an increase of at least two units or only one if the initial score was 0 (pathological condition). A worse result was defined by a decrease of two units or only one if it reaches the pathological condition (0). In the same way for BDI and STAI Y tests the cut-off was, respectively, the 85th and 40th percentile and a patient was considered improved if the score was lower than or equal to these percentiles. To make easier the interpretation of this table, the authors put in green the patients that improved their preoperative scores and in red those that did worse*.

### Matching Results

We finally matched our results to understand if there would be some predictive features for a better neurocognitive outcome. Patients with a lower Fazekas score (0–1) improved in at least 3 neurocognitive domains (above all in affective and behavioral scales and visuospatial memory), whereas the patients with preoperative higher Fazekas (2–3–4–5) had a little improvement in visuo-spatial and verbal memory and in affective and behavioral scales. One patient worsened both in affective and behavioral scales and in logical-abstract reasoning. Also, patients without preoperative compression of the sulci at the vertex seemed to do better in terms of postoperative neurocognitive outcomes in at least two domains: affective and behavioral (2 patients) and visuospatial memory (1 patient). On the other hand, patients with preoperative compression of the sulci had lower neurocognitive improvement after ETV, and, in one case, we observed impairment in three domains. Furthermore, we did not observe better postsurgical neurocognitive outcomes between the patients over or under 40 years old, as well as no difference seems to exist between the neurological and neurocognitive outcomes in the patients with chronic symptoms and the subacute onset. However, statistical analysis in a small series like this must be interpreted cautiously and has a limited value.

## Discussion

Excellent surgical results after ETV in adults affected by IAS have been reported (4, 7, 12, 25), but there are still controversies and lack of data on their neurocognitive outcomes (4, 18, 26, 27), as well as data clearly describing neuroradiological pre- and post-operative features. The aim of this study is to describe late neurocognitive outcomes in a highly homogenous series of patients.

IAS is a common cause of non-communicating hydrocephalus in childhood (6–66%), less frequent in adulthood, accounting for about 10% of all types of hydrocephalus (1, 2, 12). Other authors analyzed and discussed neuropsychological outcomes in longstanding overt ventriculomegaly in adult (LOVA) and outlined the role of ETV in the effective management of neurological and neuropsychological deficiency (28). We decided to take into consideration only patients affected by chronic adult hydrocephalus and confirmed MRI aqueductal stenosis, while, in other papers, there is a case mix. The authors consider interestingly this discussion since it is not yet clear if etiopathogeneses and symptoms of LOVA and LIAS are ascribable to a unique clinical entity (28–31). Neurological clinical classification of LIAS was firstly proposed by Fukuhara and Luciano (6). They distinguished patients with intracranial hypertension syndrome and patients experiencing NPH-like syndrome (Hakim's symptom triad) (32). Furthermore, they classified patients in relation to disease duration (chronic, subacute, and acute form). However, in their series, also patients with an incomplete or suspected stenosis of the aqueduct were included. In our series, all the patients had complete absence of CSF flow through the aqueduct in phase contrast (PC)—MRI sequences or a turbulence void signal in T2—weighted images in midsagittal thin-slice MR sequences. Eight out of 10 patients reported an iNPH-like chronic form, whereas one a subacute form. The coexistence of a headache together with NPH-like symptoms has been reported quite frequently (3, 4), but, in our series, only two patients complained of that. In our series, nearly all patients improved their clinical condition after surgical treatment; both patients with a preoperative headache became headache free as well as all the patients with gait and sphincter impairment (Table 1). These results appear to be in line with literature data (4, 12, 19, 33–39), confirming the appropriateness of the diagnostic criteria and surgical procedure, namely ETV.

While at the beginning of the twentieth century “…the difficulties encountered in treating lesions of the aqueduct of Sylvius would appear almost insuperable...” (40). Nowadays, ETV becomes the gold standard for the treatment of non-communicating hydrocephalus both in children and in adults. Many studies highlighted its safety, feasibility, low rates of intra- and postoperative complications and stable control of clinical symptoms. The rate of failure and the subsequent need for shunting are low (7, 25, 41) with a higher incidence in the pediatric population, which seems to be mostly related to age at intervention (under 6 months) (17, 42, 43).

We observed that patients' age (the symptom onset under or over 40 years old) and initial symptomatology (chronic vs. subacute form) were not related to better neurocognitive outcomes, confirming the results published by Santamarta et al. (44). After matching neuroradiological with neuropsychological pre- and post-operative results, we observed that the patients with lower Fazekas scores (lower cortical, subcortical, and periventricular hyperintensity), as well as the patients without preoperative compression of the sulci at the vertex, seemed to achieve improvement in their preoperative neurocognitive impairments even if data are not supported by statistical analysis, probably due to the small population size.

Controversies also regarding postoperative reduction in ventricular size and a favorable neurological and cognitive outcome exist. While some studies support this hypothesis (33, 45, 46), in particular, the reduction of the third ventricle size (33, 44, 47), other studies have failed to confirm this correlation, concluding that the ventricles' size is not a valid predictor of clinical and intellectual outcomes, and reliance on imaging should be avoided (38, 48, 49). In our series, Evan's ratio decreased only in two cases, while temporal horns reduction was found in six out of 10 patients. This reduction was not related to the clinical outcome (all the patients improved regardless of the ventricular size) nor to the neurological outcome. Similar findings were reported by Rodis et al. (50) in a retrospective outcome analysis where they found that only 5% of patients with LIAS reduced Evan's ratio after ETV.

Analyzing the compression of cranial subarachnoid convexity spaces (namely, vertex sulci and parafalcine sulci) (51, 52), we recorded their normalization in all cases with a good concordance of neurological improvement. These findings could reflect the fact that, as suggested by Tisell et al. (53), connections of two CSF compartments (intraventricular and subarachnoid spaces—SAS) decrease the resistance to the outflow of CSF (Rout), increasing the area of CSF absorption in both the SAS and the ventricles.

In our opinion, interpeduncular height could represent an indirect sign of normalization of the difference of pressure among intraventricular areas and SAS at the level of the cranial base (Figure 2). It seems to be a reliable finding since all the patients that improved neurologically had an increase of postoperative interpeduncular height. This indirect sign of third ventricle/hypothalamus relaxation corroborates Larsson's suggestion of an increased regional cerebral blood flow in the upper brain stem, hippocampi, and frontal region after shunting the procedure in iNPH (54). However, increasing interpeduncular height is related to neurological improvement but does not appear to be directly related to a neurocognitive outcome.

We found a significant improvement of attentive and executive functions as well as visuo-spatial memory and verbal executive functions after ETV, and, similarly, we observed improvements in the affective and behavioral scales (specifically assessing the rate of depression and anxiety) in almost 50% of the patients (*p* <0.05, Table 4).

Our extensive neuropsychological analysis (see Table 5) in a highly homogeneous cohort of patients supports the hypothesis suggested by Burtscher and Hader (18, 19) that ETV may improve neurological symptoms and intellectual functions in patients with LIAS. In our experience, neurocognitive evaluation has proved to be a useful tool to define preexisting impairments due to hydrocephalus and to discriminate them from any postoperative findings (55).

Interestingly, we observed that all the patients had a normal MMSE, indeed a not typical finding in chronic late-onset hydrocephalus, suggesting that a deep neurocognitive evaluation is mandatory in these patients.

Furthermore, we recommend neurocognitive analysis in patients with LIAS to better elucidate the natural history of this syndrome. Indeed, many of the up-to-now so-called “asymptomatic” patients affected by aqueductal stenosis did not undergo extensive neuropsychological evaluation, preventing us from knowing if “neglected” intellectual deficits may be present or may even represent the first onset. This knowledge could better define a correct follow-up (neuroradiological, clinical, and neurocognitive) and a well-timed surgical treatment also in these cases.

## Limitations

This is a small case series of prospectively collected data. Only limited statistical analysis was performed due to the sample size. Our preliminary results thus need further confirmation in wider series and case-control studies. In addition, the postoperative neurocognitive evaluations were performed during a wide time span after surgery, and this represents a significant limitation of their value.

## Conclusion

Endoscopic third ventriculostomy in patients with LIAS seems to be an effective treatment, improving both neurological and neurocognitive outcomes. Further studies are warranted to better clarify the complex neurocognitive analysis as well as LIAS natural history.

## Data Availability Statement

The original contributions presented in the study are included in the article/supplementary material, further inquiries can be directed to the corresponding author/s.

## Ethics Statement

The studies involving human participants were reviewed and approved by the Local Ethics Committee of the Local Health Service of Bologna, Italy. The patients/participants provided their written informed consent to participate in this study.

## Author Contributions

MM and GP participated to ideate, write, and revise the manuscript. GM took part to write the draft. LR, FS, GB, CZ, DM, AC, and LC took part to the revision process. All authors contributed to the article and approved the submitted version.

## Conflict of Interest

The authors declare that the research was conducted in the absence of any commercial or financial relationships that could be construed as a potential conflict of interest.

## Publisher's Note

All claims expressed in this article are solely those of the authors and do not necessarily represent those of their affiliated organizations, or those of the publisher, the editors and the reviewers. Any product that may be evaluated in this article, or claim that may be made by its manufacturer, is not guaranteed or endorsed by the publisher.
